# Supplementary material for: Estimating the generation time for influenza transmission using household data in the United States
Source: Epidemics. Author manuscript; Available in PMC 2025 Apr 11. (PMC11986874; doi:10.1016/j.epidem.2025.100815)
Supplement: 1 [file NIHMS2066144-supplement-1.docx]

# Supplementary material

## The household data across vaccination status, age group and study site

The household data used in the primary analysis, which excludes households with multiple co-primary cases, comprised 820 individuals: 246 primary cases from 246 households and 574 household contacts, of which 274 tested negative for influenza and remained uninfected. Supplemental Table S1 presents the characteristics of the household data by vaccination status, age group, and study site.

| **Data stratifications** | **Number of individuals (primary cases)** | **Symptomatic infected household members %** | **Asymptomatic infected household members %** | **Uninfected household members %** |
| --- | --- | --- | --- | --- |
| All data excluding households with multiple co-primary cases (primary analysis) | 820 (246) | 59.4% (487/820) | 7.2% (59/820) | 33.4% (274/820) |
| **By vaccination status** | | | | |
| - Vaccinated | 300 (94) | 57.0% (171/300) | 7.3% (22/300) | 35.7% (107/300) |
| - Unvaccinated | 477 (138) | 60.8% (290/477) | 7.6% (26/477) | 31.7% (151/477) |
| - Unknown | 43 (14) | 60.5% (26/43) | 2.3% (1/43) | 37.2% (16/43) |
| **By age group (years)** | | | | |
| - 0-4 | 86 (43) | 84.9% (73/86) | 3.5% (3/86) | 11.6% (10/86) |
| - 5-11 | 181 (89) | 71.3% (129/181) | 6.6% (12/181) | 22.1% (40/181) |
| - 12-17 | 123 (52) | 61.8% (76/123) | 7.3% (9/123) | 30.9% (38/123) |
| - 18-49 | 343 (43) | 48.1% (165/343) | 7.3% (25/343) | 44.6% (153/343) |
| - 50-64 | 59 (12) | 44.1% (26/59) | 16.9% (10/59) | 39.0% (23/59) |
| - 65+ | 28 (7) | 64.3% (18/28) | 0% (0/28) | 35.7% (10/28) |
| **By study site** | | | | |
| - University of Arizona | 12 (3) | 66.7% (8/12) | 0% (0/12) | 33.3% (4/12) |
| - University of Colorado School of Medicine and Children’s Hospital Colorado | 3 (1) | 100% (3/3) | 0% (0/3) | 0% (0/3) |
| - Columbia University Irving Medical Center | 406 (115) | 56.4% (229/406) | 8.9% (36/406) | 34.7% (141/406) |
| - Marshfield Clinic Research Institute | 26 (8) | 61.5% (16/26) | 0% (0/26) | 38.5% (10/26) |
| - Vanderbilt University Medical Center | 312 (101) | 60.6% (189/312) | 7.1% (22/312) | 32.4% (101/312) |
| - Stanford University | 20 (6) | 80.0% (16/20) | 0% (0/20) | 20.0% (4/20) |
| - University of North Carolina at Chapel Hill | 41 (12) | 63.4% (26/41) | 2.4% (1/41) | 34.1% (14/41) |

Table S1. Characteristics of household data. The first row presents the primary analysis, along with three data stratifications by vaccination status, age group, and study site, which all excluded households with multiple co-primary cases.

## The observed household serial interval of single infection pairs

We found that the observed household serial interval, calculated without modeling, solely using data from households with single infection pairs (i.e., single primary case to single secondary case) and without potential transmission chains, had a mean of 3.7 days (and a SD of 2.3 days). This was longer than the mean intrinsic serial interval of 3.2 (95% CrI: 2.8-3.5) days when considering households of all sizes with all potential transmission chains (Table 2). This does not necessarily indicate that the intrinsic value was shorter than the realized household one. Rather, it is mainly due to the restriction of single infection pairs or mostly smaller household sizes of 2 members.

In the main text, we found slightly longer mean intrinsic and realized household generation times in smaller households compared to larger ones (Figure 1C and Supplemental Table S2). Larger households with more exposure and potential transmission chains could have a shorter interval, while smaller households could have a longer interval.

## Specification of parameters for the mechanistic model

The mechanistic SEIR model includes compartments for susceptible (S), latent (E), asymptomatic infectious (A), pre-symptomatic infectious (P), symptomatic infectious (I), and recovered (R) stages.

The latent period $y_{E}$ and pre-symptomatic infectious period $y_{P}$ are assumed to follow gamma distributions, $y_{E}\sim\text{Gamma}\left( k_{E},\frac{1}{\left( k_{E}+k_{P} \right)\gamma} \right)$ and $y_{P}\sim\text{Gamma}\left( k_{P},\frac{1}{\left( k_{E}+k_{P} \right)\gamma} \right)$. The corresponding mean periods are $\frac{k_{E}}{\left( k_{E}+k_{P} \right)\gamma}$ and $\frac{k_{P}}{\left( k_{E}+k_{P} \right)\gamma}$, respectively. Thus, the incubation period $\tau_{inc}=y_{E}+y_{P}$ (the sum of latent and pre-symptomatic infectious period) is also gamma distributed, $\tau_{inc}\sim\text{Gamma}\left( k_{inc},\frac{1}{k_{inc}\gamma} \right)$, where $k_{inc}=k_{E}+k_{P}$ is the shape parameter, and $\frac{1}{\gamma}$ represents the mean incubation period. The symptomatic infectious period $y_{I}$ is assumed to follow an exponential distribution, $y_{I}\sim\text{Exp}\left( \mu\right)$, where $\frac{1}{\mu}$ is the mean symptomatic infectious period.

The force of infection $\beta\left( \tau\right)$ of an infected individual at the time since infection $\tau$ is modeled as $\beta\left( \tau\right)=\alpha\frac{\beta_{0}}{n}f\left( \tau\right)$, where $\beta_{0}$ is the overall infectiousness (referring to the expected number of household transmissions generated by a single symptomatic infected primary case), $n$ is the household size, $f\left( \tau\right)$ is the generation time distribution, and $\alpha$ is the relative infectiousness in various infectious stages. Further details of the mechanistic model can be found in the original study (Hart, Abbott, et al. 2022).

We assumed $\alpha_{I}=1$ for symptomatic infectious individuals and $\alpha_{A}=0.57$ (0.11 or 1.54 in the sensitivity analysis) for asymptomatic infectious individuals (Tsang, et al. 2023). We estimated $\alpha_{P}$ for pre-symptomatic infectious individuals (representing the ratio of pre-symptomatic and symptomatic transmission rates), along with three additional parameters: the ratio of the mean latent and incubation period $\frac{k_{E}}{k_{inc}}$ (where $k_{inc}$ is assumed to be known and estimated separately), the mean symptomatic infectious period $\frac{1}{\mu}$ and the overall infectiousness $\beta_{0}$. These parameters $\theta=\left( \frac{k_{E}}{k_{inc}},\frac{1}{\mu},\alpha_{P},\beta_{0} \right)$ were estimated using the Bayesian data augmentation MCMC method. The distributions of the four estimated parameters are illustrated in Supplemental Figure S1.

The proportion of transmission before symptomatic onset (Table 3) is calculated as $\frac{\alpha_{P}\frac{k_{P}}{k_{inc}\gamma}}{\alpha_{P}\frac{k_{P}}{k_{inc}\gamma}+\frac{1}{\mu}}$. This is determined by weighting the pre-symptomatic period $\frac{k_{P}}{k_{inc}\gamma}$ by the ratio of pre-symptomatic and symptomatic transmission rates $\alpha_{P}$, and then dividing it by the total infectious period (the sum of the pre-symptomatic period $\alpha_{P}\frac{k_{P}}{k_{inc}\gamma}$ and the symptomatic period $\frac{1}{\mu}$).


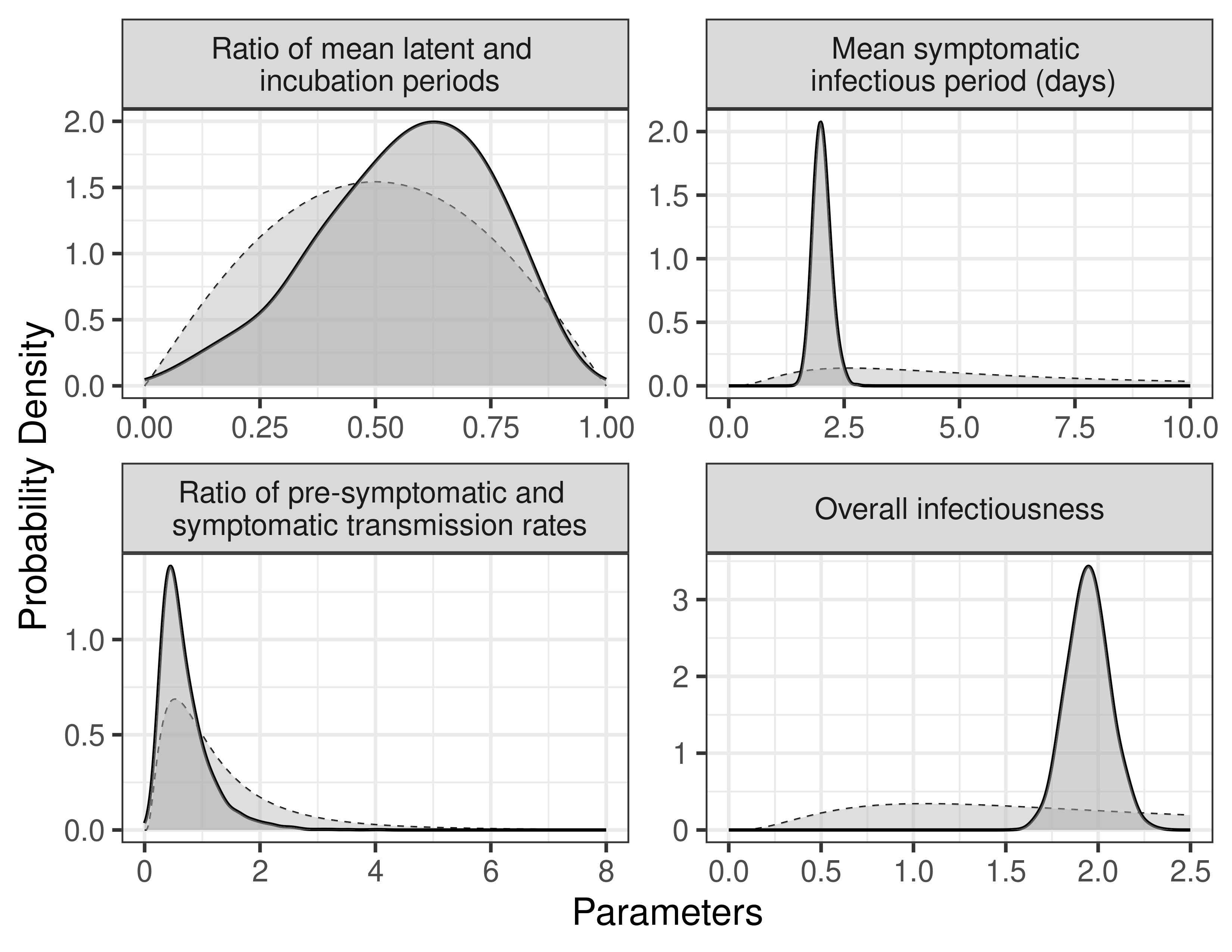


Figure S1. Posterior and prior distributions of the estimated parameters $\theta=\left( \frac{k_{E}}{k_{inc}},\frac{1}{\mu},\alpha_{P},\beta_{0} \right)$ in the primary analysis. The solid and dashed lines represent the posterior and prior distributions, respectively.

## The influenza-specific incubation period distribution

The two incubation period parameters ($k_{inc}$ and $\gamma$) were estimated by fitting a gamma distribution to estimates for influenza A from a systematic review by Lessler et al. (Lessler, et al. 2009). We found the shape parameter $k_{inc}$ to be 5.60 and the scale parameter to be 0.28 (equivalent to $\gamma=0.64$). The corresponding mean and standard deviation (SD) are 1.55 days and 0.66 days, respectively. The incubation period distribution is illustrated in Supplemental Figure S2.


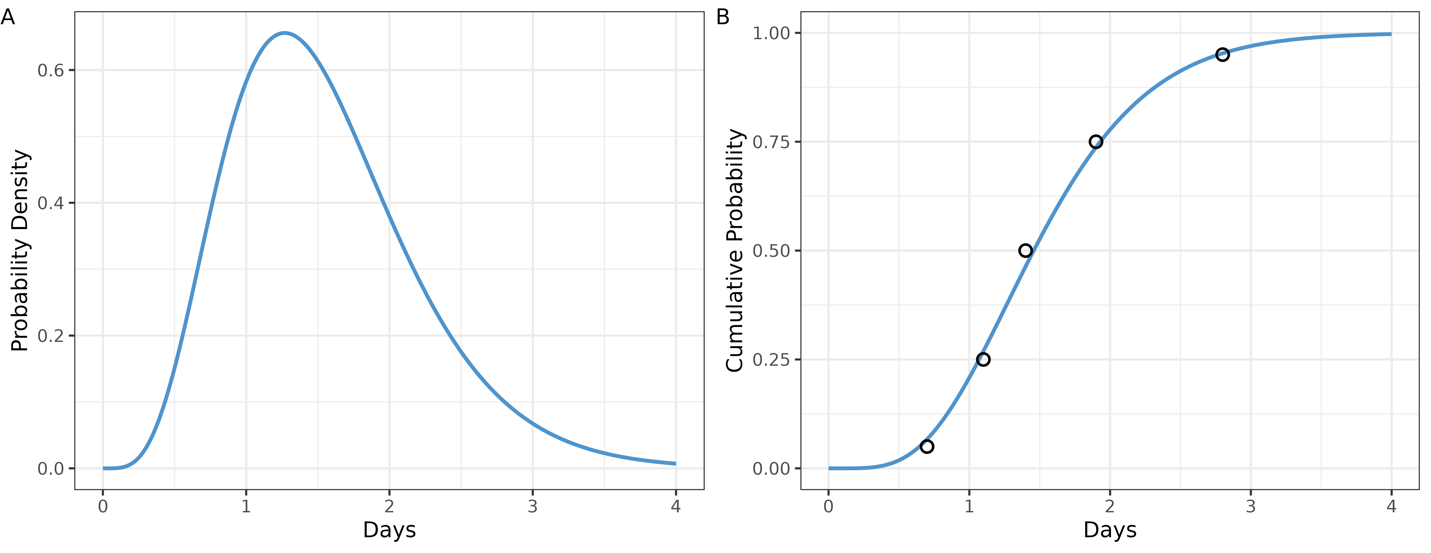


Figure S2. Incubation period distribution. The black circles and blue lines represent the data (Lessler, et al. 2009), and the (A) cumulative distribution function and (B) probability density function of a gamma distribution fitted to the data.

## The likelihood function

For a household of size $n$, the likelihood function consists of two parts: the contribution from transmission $L_{k,1}\left( D | \theta\right)$, and the incubation period $L_{k,2}\left( D | \theta\right)$ for each individual $k$, given parameter $\theta$ and data $D$. The overall likelihood is expressed as $L\left( D | \theta\right)=\prod_{k=1}^{n} L_{k,1}\left( D | \theta\right)L_{k,2}\left( D | \theta\right)$.

In the first part, for a primary case $k$, the likelihood of transmission is set to $L_{k,1}\left( D | \theta\right)=1$. For an infected individual $k$ with infection time $t_{k}$, the likelihood of transmission is given by $L_{k,1}\left( D | \theta\right)=\lambda\left( t_{k} \right)e^{-\int_{-\infty}^{t_{k}} \lambda\left( s \right)\mathrm{ds}}$, where the total force of infection is $\lambda\left( t_{k} \right)=\sum_{j} \beta\left( t_{k}-t_{j} \right)$ and $t_{j}$ is the infection time of other earlier infected household members $j$. For an uninfected individual $k$, taking $t_{k}=+\infty$, the likelihood of transmission becomes $L_{k,1}\left( D | \theta\right)=e^{-\int_{-\infty}^{+\infty} \lambda\left( s \right)\mathrm{ds}}$.

In the second part, for an infected individual $k$, the likelihood of the incubation period is given by $L_{k,2}\left( D | \theta\right)=f_{inc}\left( \eta_{k}-t_{k} \right)$, where $f_{inc}\left( \eta_{k}-t_{k} \right)$ represents the incubation period distribution and $\eta_{k}$ is the time of symptom onset for individual $k$. For an uninfected individual $k$, the likelihood of the incubation period is set to $L_{k,2}\left( D | \theta\right)=1$.

Further details of the likelihood function can be found in the original study (Hart, Abbott, et al. 2022).

## The Bayesian data argumentation Markov Chain Monte Carlo (MCMC)

The Bayesian data augmentation MCMC method involves estimating model parameters and augmenting observed data with infection times and symptom onset times. Each iteration of the MCMC chain consists of four key steps. (1) New model parameters are proposed using a multivariate normal proposal distribution. (2) Precise times of symptom onset of each symptomatic infected individuals are proposed using independent uniform proposal distributions. (3) Precise times of infection of symptomatic infected individuals are proposed using independent normal proposal distributions. (4) Precise times of infection of asymptomatic infected individuals are proposed using independent normal proposal distributions.

For each step, the proposed values are accepted based on the acceptance probability, which is the ratio of the proposed posterior to the current posterior. The posterior is the product of the likelihood and the prior distribution. For last three steps, the acceptance probability simplifies to the ratio of the proposed likelihood to the current likelihood. If the ratio is greater than 1, the proposed values are accepted, i.e., acceptance probability is 1.

Further details of the Bayesian data augmentation MCMC method can be found in the original study (Hart, Abbott, et al. 2022).

## Variability in estimates across data stratifications

Although the generation time or serial interval of influenza B may be longer than that of influenza A (Levy, et al. 2013), this was not the case in our findings from the two seasons (Supplemental Table S2, Figure S3 and S4). However, we note that the mean intrinsic generation time exhibited a wider credible interval when using data exclusively from influenza B compared to influenza A, which likely reflects the dominance of influenza A during the study timeframe and the smaller sample size of influenza B.

| **Data stratifications** | **Mean intrinsic generation time (95% CrIs)** | **Overlapping index (%, compared to the primary analysis)** |
| --- | --- | --- |
| All data excluding households with multiple co-primary cases (primary analysis in Table 1) | 3.2 (2.9-3.6) | 100 |
| Season 2021/2022 | 3.3 (2.8-4.0) | 71 |
| Season 2022/2023 | 3.2 (2.8-3.6) | 87 |
| Influenza A | 3.2 (2.9-3.6) | 94 |
| Influenza B | 3.2 (2.3-4.5) | 47 |
| Household size of 2 or 3 | 3.4 (2.9-4.0) | 61 |
| Household size of 4 or greater | 3.1 (2.7-3.6) | 74 |
| All data including households with multiple co-primary cases | 3.1 (2.7-3.4) | 64 |

Table S2. The posterior mean (95% CrIs) of the mean intrinsic generation time across seasons, virus types, household sizes, and with multiple co-primary cases. The incubation period, derived from influenza A, had a mean of 1.55 days and a standard deviation (SD) of 0.66 days (Lessler, et al. 2009). Only for influenza B, we assumed the shorter incubation period to yield a mean of 0.61 days and a standard deviation (SD) of 0.25 days.


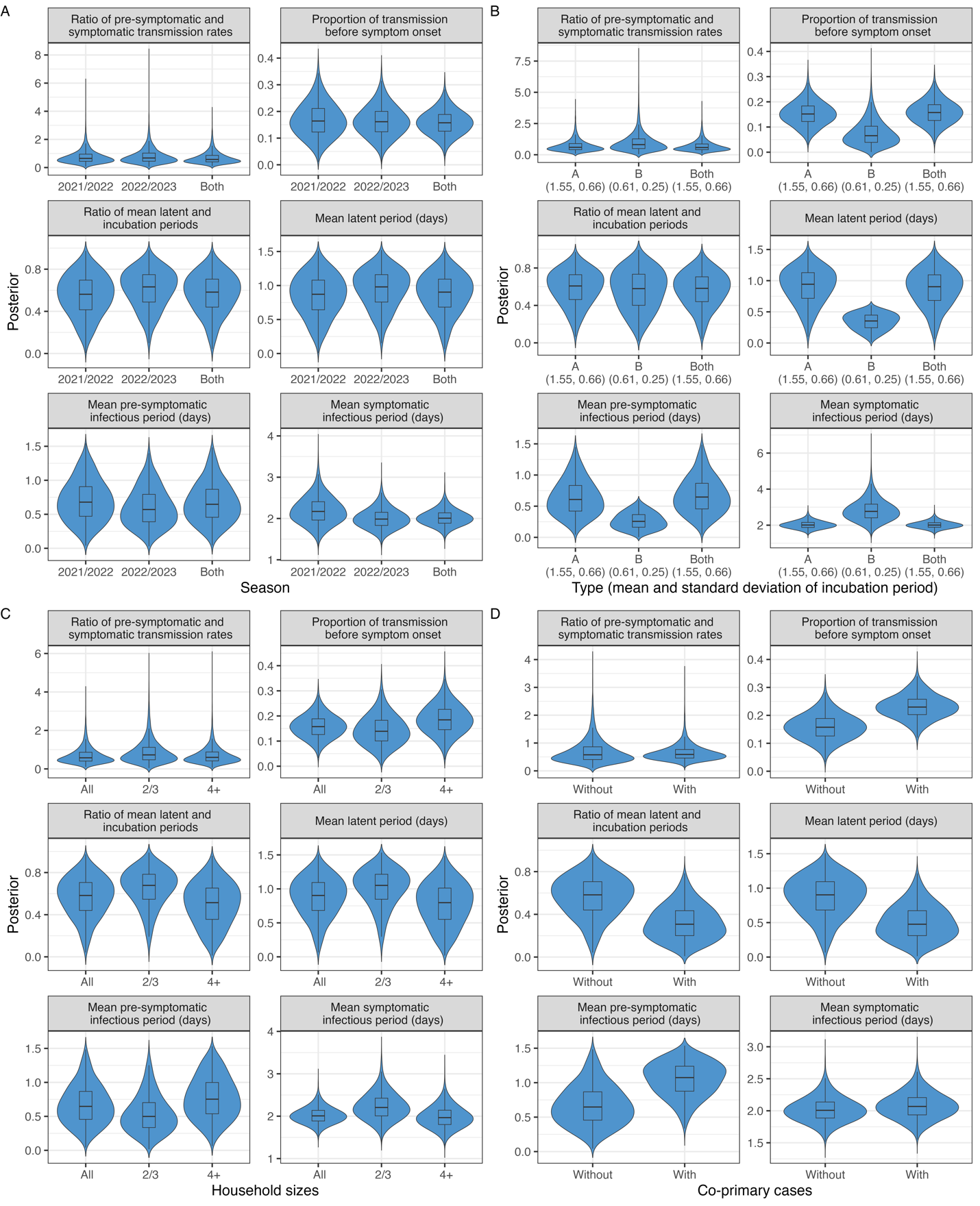


Figure S3. Posterior distributions of the parameters across data stratifications: (A) seasons, (B) virus types, (C) household sizes, and (D) with multiple co-primary cases.


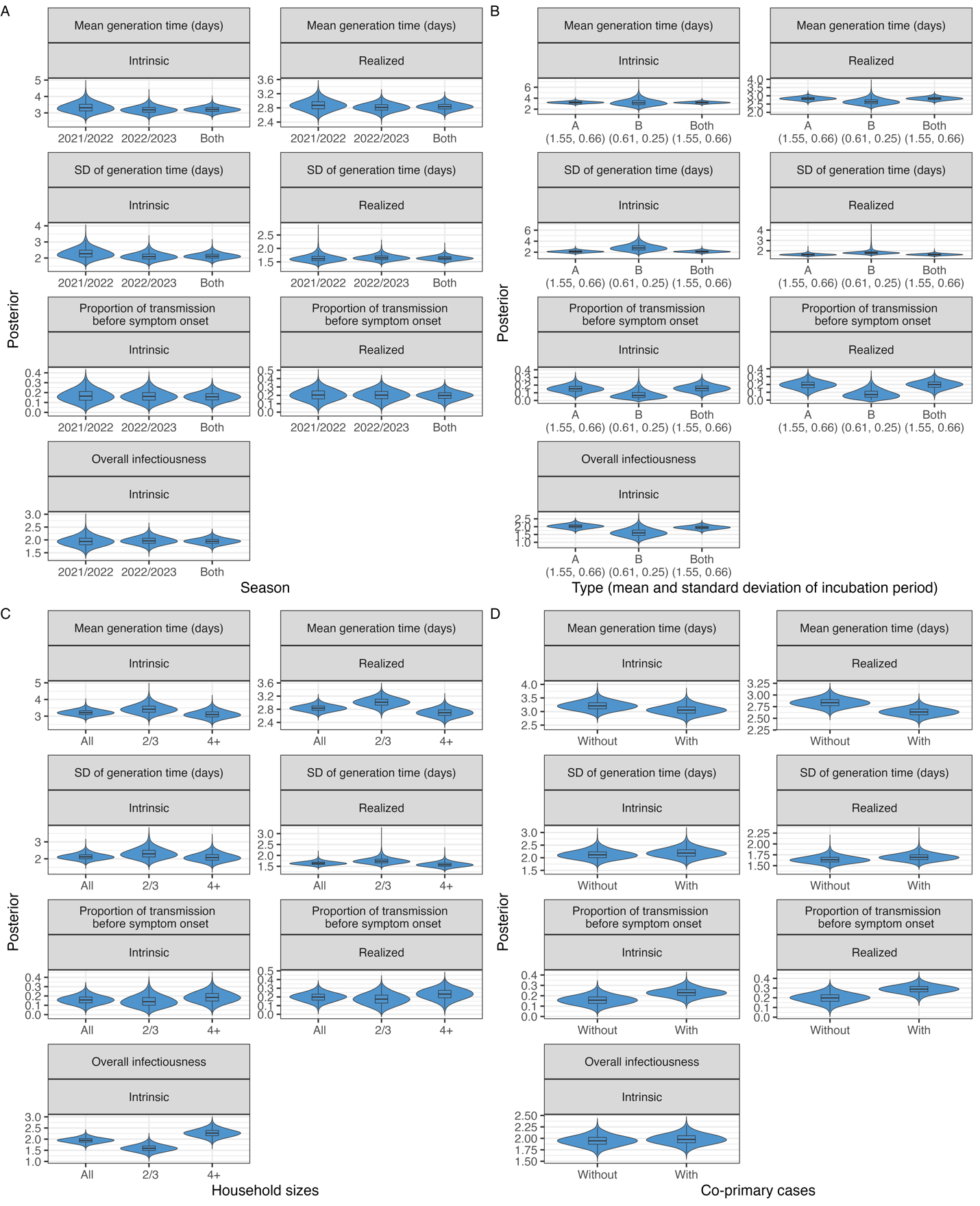


Figure S4. Posterior distributions of the parameters across data stratifications: (A) seasons, (B) virus types, (C) household sizes, and (D) with multiple co-primary cases.

## Estimates of the generation time by vaccination status

We estimated the mean intrinsic generation time by vaccination status of primary cases and their household contacts (Table S3), in addition to stratifying the data across seasons, virus types, and household sizes (Table S2). First, we stratified the data based on the vaccination status of primary cases (vaccinated, unvaccinated, or unknown), considering all their household contacts. Then, we analyzed the data based on the vaccination status of household contacts, who were categorized as vaccinated, unvaccinated, or a mixture (including vaccinated, unvaccinated, and unknown statuses).

There were 94 households with vaccinated primary cases, 138 households with unvaccinated primary cases, and 14 households with primary cases of unknown vaccination status. On the other hand, there were 50 households where all contacts were vaccinated, 116 households where no contacts were vaccinated, and 80 households with a mixture of statuses (vaccinated, unvaccinated, and unknown).

We found that the vaccination status of primary cases and their household contacts did not significantly affect the mean intrinsic generation time estimates, which were similar for vaccinated primary cases (3.3 days; 95% CrI: 2.7, 3.9) and unvaccinated primary cases (3.3 days; 95% CrI: 2.9, 3.8). Similarly, the estimates for vaccinated household contacts (3.5 days; 95% CrI: 2.7, 4.7) and unvaccinated household contacts (3.5 days; 95% CrI: 3.0, 4.0) were comparable, although the unknown statuses or mixture groups exhibited slightly shorter estimates.

We also estimated the overall infectiousness, which refers to the expected number of household transmissions generated by a single symptomatic infected primary case. The mean overall infectiousness was slightly lower among vaccinated individuals, whether they were primary cases or household contacts, compared to unvaccinated individuals. Notably, both vaccinated and unvaccinated groups shared overlapping credible intervals. It is important to note that this analysis does not explicitly distinguish between differences in susceptibility or infectivity by vaccination status.

When analyzing the combined vaccination status of primary cases and their household contacts, the estimates varied across different combinations. However, wide and overlapping credible intervals suggest greater uncertainty due to smaller sample sizes (N). For example, the mean intrinsic generation time for vaccinated primary cases with vaccinated household contacts (N=36) was estimated at 3.8 days (95% CrI: 2.8, 5.1), while unvaccinated primary cases with unvaccinated household contacts (N=95) had an estimate of 3.4 days (95% CrI: 2.9, 4.0).

Overall, the findings suggest that vaccination status has a limited effect on the intrinsic generation time.

| **Vaccination status of primary cases (N)** | **Vaccination status of household contacts (N)** | **Mean intrinsic generation time (95% CrIs)** | **Overall infectiousness (95% CrIs)** |
| --- | --- | --- | --- |
| **By vaccination status of primary cases** | | | |
| Vaccinated (94) | All (236) | 3.3 (2.7, 3.9) | 1.9 (1.6, 2.3) |
| Unvaccinated (138) | All (302) | 3.3 (2.9, 3.8) | 2.0 (1.7, 2.4) |
| Unknown (14) | All (36) | 3.0 (2.0, 4.8) | 1.6 (0.9, 2.4) |
| **By vaccination status of household contacts** | | | |
| All (50) | Vaccinated (96) | 3.5 (2.7, 4.7) | 1.9 (1.4, 2.5) |
| All (116) | Unvaccinated (244) | 3.5 (3.0, 4.0) | 2.2 (1.8, 2.5) |
| All (80) | Mixture (vaccinated/unvaccinated/unknown) (234) | 2.9 (2.4, 3.4) | 1.8 (1.5, 2.1) |
| **By vaccination status of both primary cases and household contacts** | | | |
| Vaccinated (36) | Vaccinated (78) | 3.8 (2.8, 5.1) | 2.3 (1.7, 3.1) |
| Vaccinated (18) | Unvaccinated (33) | 4.2 (2.8, 6.4) | 2.1 (1.3, 3.1) |
| Vaccinated (40) | Mixture (vaccinated/unvaccinated/unknown) (125) | 2.7 (2.1, 3.5) | 1.7 (1.2, 2.1) |
| Unvaccinated (13) | Vaccinated (16) | 3.6 (1.7, 8.2) | 0.9 (0.4, 1.7) |
| Unvaccinated (95) | Unvaccinated (203) | 3.4 (2.9, 4.0) | 2.2 (1.8, 2.6) |
| Unvaccinated (30) | Mixture (vaccinated/unvaccinated/unknown) (83) | 3.4 (2.7, 4.4) | 2.2 (1.6, 2.9) |
| Unknown (1) | Vaccinated (2) | 6.5 (2.1, 18.2) | 1.6 (0.4, 3.9) |
| Unknown (3) | Unvaccinated (8) | 4.5 (1.9, 10.3) | 2.4 (1.0, 4.8) |
| Unknown (10) | Mixture (vaccinated/unvaccinated/unknown) (26) | 3.0 (1.8, 5.5) | 1.4 (0.7, 2.3) |

Table S3. The posterior mean (95% CrIs) of the mean intrinsic generation time and overall infectiousness by vaccination status of primary cases and household contacts, and the combination of both primary cases and household contacts. The overall infectiousness refers to the expected number of household transmissions generated by a single symptomatic infected primary case.

## Sensitivity analyses

Similar to the sensitivity analyses using the full dataset (Supplemental Figure S6, Panel A), we found that the incubation period had a limited effect on the intrinsic generation time when exclusively using data from households circulating influenza A (Supplemental Figure S6, Panel B) or households circulating influenza B (Supplemental Figure S6, Panel C).

Consistent with the previous study (Hart, Abbott, et al. 2022), assuming a higher relative infectiousness of asymptomatic infected individuals resulted in slightly lower estimates of the overall infectiousness of infectors (Supplemental Figure S6, Panel D).

| **Sensitivity analyses** | **Mean intrinsic generation time (95% CrIs)** | **Overlapping index (%, compared to the primary analysis)** |
| --- | --- | --- |
| Primary analysis (in Table 1) | 3.2 (2.9-3.6) | 100 |
| Longer incubation period | 3.2 (2.8-3.6) | 86 |
| Shorter incubation period | 3.4 (3.1-3.7) | 56 |
| Lower relative infectiousness | 3.2 (2.9-3.6) | 94 |
| Higher relative infectiousness | 3.2 (2.9-3.6) | 97 |

Table S4. The posterior mean (95% CrIs) of the mean intrinsic generation time given different incubation periods or relative infectiousness of asymptomatic infected individuals. The primary incubation period, derived from influenza A, had a mean of 1.55 days and a standard deviation (SD) of 0.66 days (Lessler, et al. 2009). For the shorter incubation period derived from influenza B, we assumed a mean of 0.61 days and a SD of 0.25 days (Lessler, et al. 2009). For the longer incubation period derived from influenza A(H1N1)pdm09, we assumed a mean of 4.30 days and a SD of 1.25 days (Tuite, et al. 2010).


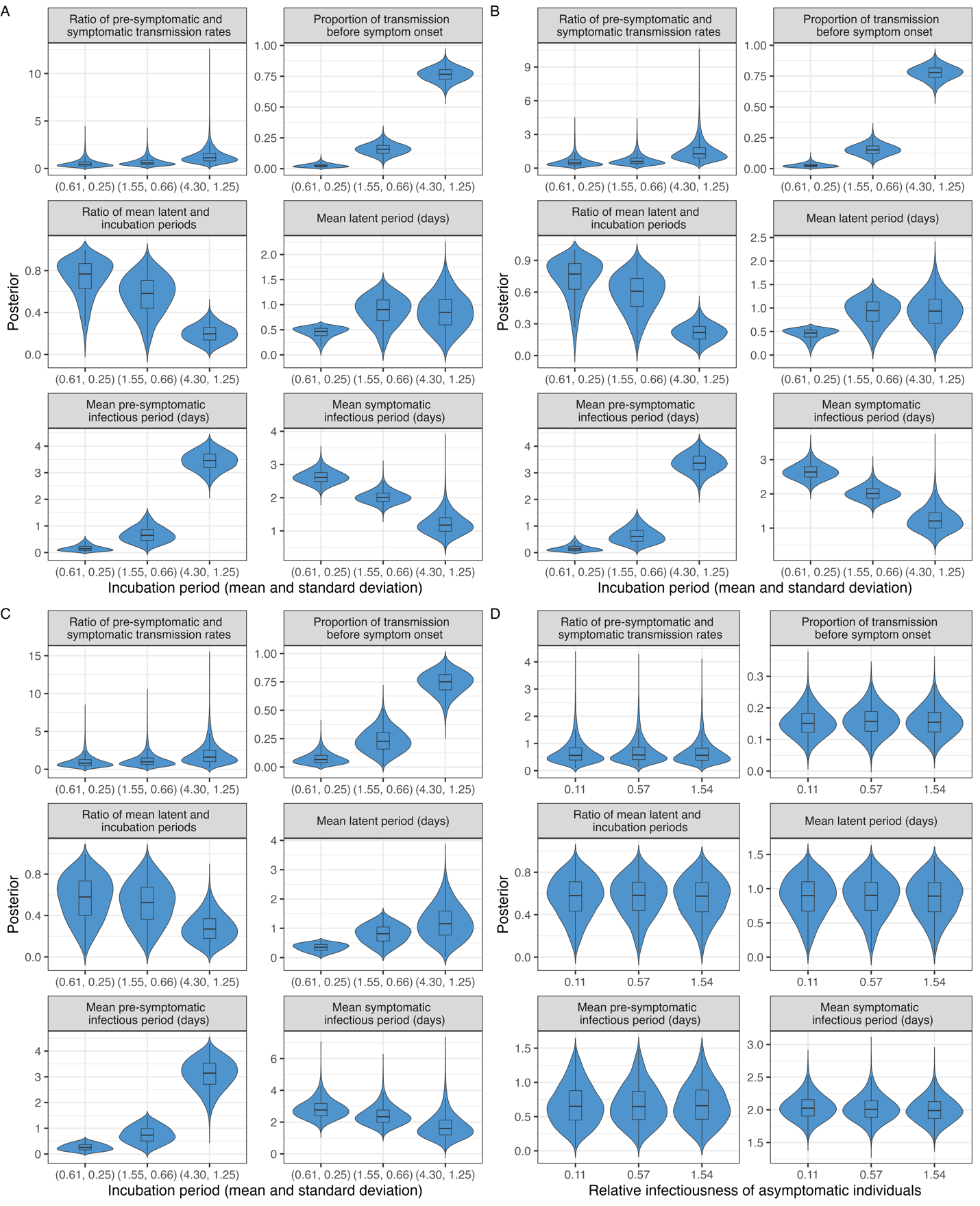


Figure S5. Posterior distributions of the parameters given different assumptions: (A-C) incubation periods, and (D) relative infectiousness of asymptomatic infected individuals. Panel (A) presents results obtained using data from households with both influenza A and B, whereas Panels (B) and (C) present results obtained using data solely from households with influenza A and B, respectively.


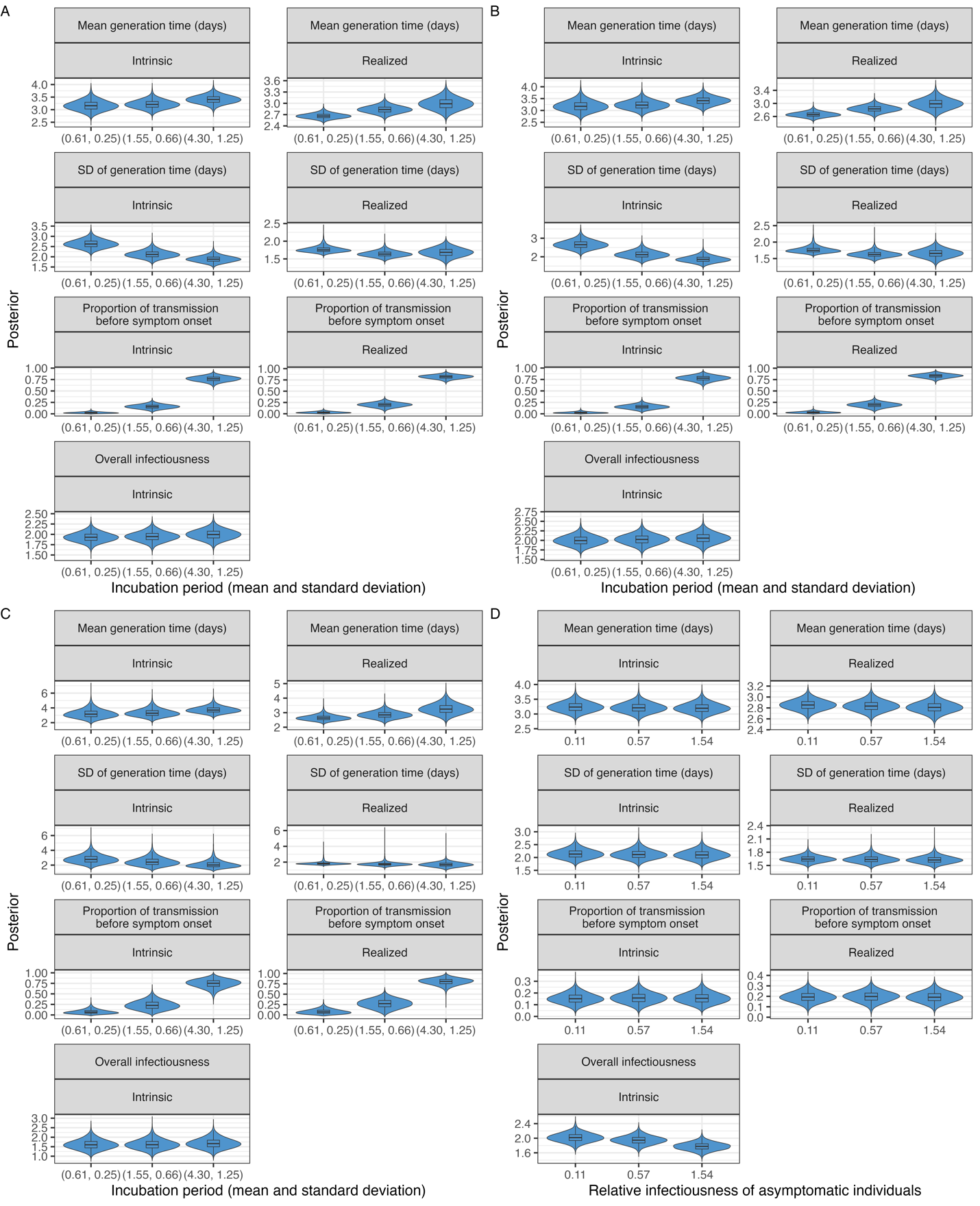


Figure S6. Posterior distributions of the parameters given different assumptions: (A-C) incubation periods, and (D) relative infectiousness of asymptomatic infected individuals. Panel (A) presents results obtained using data from households with both influenza A and B, whereas Panels (B) and (C) present results obtained using data solely from households with influenza A and B, respectively.
